# Beyond PKA: Evolutionary and structural insights that define a docking and dimerization domain superfamily

**DOI:** 10.1016/j.jbc.2021.100927

**Published:** 2021-07-10

**Authors:** Heather R. Dahlin, Ning Zheng, John D. Scott

**Affiliations:** 1 Department of Pharmacology, University of Washington, Seattle, Washington, USA; 2 Howard Hughes Medical Institute, University of Washington, Seattle, Washington, USA

**Keywords:** PKA, docking and dimerization (D/D) domain, A-kinase-anchoring protein (AKAP), heterodimerization, cell signaling, AKAPs, A-kinase-anchoring proteins, D/D, docking and dimerization, DPY-30, Dumpy-30, MALS, multiangle light scattering, PKA-R, PKA regulatory, SEC, size-exclusion chromatography, SEC-MALS, size-exclusion chromatography coupled to multiangle light scattering, SPA17, sperm autoantigenic protein 17

## Abstract

Protein-interaction domains can create unique macromolecular complexes that drive evolutionary innovation. By combining bioinformatic and phylogenetic analyses with structural approaches, we have discovered that the docking and dimerization (D/D) domain of the PKA regulatory subunit is an ancient and conserved protein fold. An archetypal function of this module is to interact with A-kinase-anchoring proteins (AKAPs) that facilitate compartmentalization of this key cell-signaling enzyme. Homology searching reveals that D/D domain proteins comprise a superfamily with 18 members that function in a variety of molecular and cellular contexts. Further *in silico* analyses indicate that D/D domains segregate into subgroups on the basis of their similarity to type I or type II PKA regulatory subunits. The sperm autoantigenic protein 17 (SPA17) is a prototype of the type II or R2D2 subgroup that is conserved across metazoan phyla. We determined the crystal structure of an extended D/D domain from SPA17 (amino acids 1–75) at 1.72 Å resolution. This revealed a four-helix bundle-like configuration featuring terminal β-strands that can mediate higher order oligomerization. In solution, SPA17 forms both homodimers and tetramers and displays a weak affinity for AKAP18. Quantitative approaches reveal that AKAP18 binding occurs at nanomolar affinity when SPA17 heterodimerizes with the ropporin-1-like D/D protein. These findings expand the role of the D/D fold as a versatile protein-interaction element that maintains the integrity of macromolecular architectures within organelles such as motile cilia.

Protein–protein interactions constrain macromolecules to form molecular machines (1). A-kinase-anchoring proteins (AKAPs) confine PKA within ‘signaling islands’ to create highly organized signaling compartments (2, 3, 4). A defining attribute of AKAPs is an amphipathic α-helix that binds with high affinity to the docking and dimerization (D/D) domain of PKA regulatory (PKA-R) subunits (5, 6). The PKA holoenzyme is composed of two catalytic (C) subunits constrained by an R subunit dimer (7, 8). This broad-spectrum kinase and the signaling events that it influences have been a focus of research spanning over 65 years (9, 10, 11). The R-subunit is composed of an amino-terminal D/D domain connected *via* an autoinhibitory or pseudosubstrate linker to tandem cAMP-binding pockets. There are four known isoforms of PKA-R subunits: RIα, RIβ, RIIα, and RIIβ (8). Each gene arose from duplication and expansion from an ancestral R-subunit. These PKA–R-subunit isoforms display distinct physiochemical properties and exhibit differential binding affinities for AKAPs (2, 4, 12).

The D/D domain was discovered as a unique region of PKA–RIIα subunit that mediates dimerization and AKAP binding (13). Subsequent NMR and crystallographic studies have characterized the structure of the RI and RII D/D domains in complex with AKAPs (14, 15). R-subunit protomers dimerize to form an X-type helix bundle in an antiparallel arrangement (Fig. 1*A*). A hydrophobic groove formed at the top of this substructure docks with an amphipathic α-helix on the surface of the AKAP (16, 17, 18). A helical segment at the amino terminus of RI subunits orients key determinants for AKAP binding (Fig. 1*A*). In RII subunits, the first five amino acids form β-strands that are essential for docking, with isoleucine’s 3 and 5 serving as key PKA-anchoring determinants (19) (Fig. 1*A*). About 60 AKAPs have been identified, each containing a PKA-anchoring helix that associates with D/D domains (Omar and Scott, 2020). These regions of secondary structure have degenerate sequences of 14 to 18 residues, but with a discernable pattern of hydrophobic amino acids critical for docking (5, 12). Peptide studies have uncovered primary structure determinants that influence AKAP binding to RI and to RII (6, 20, 21, 22, 23, 24, 25).

**Figure 1:**
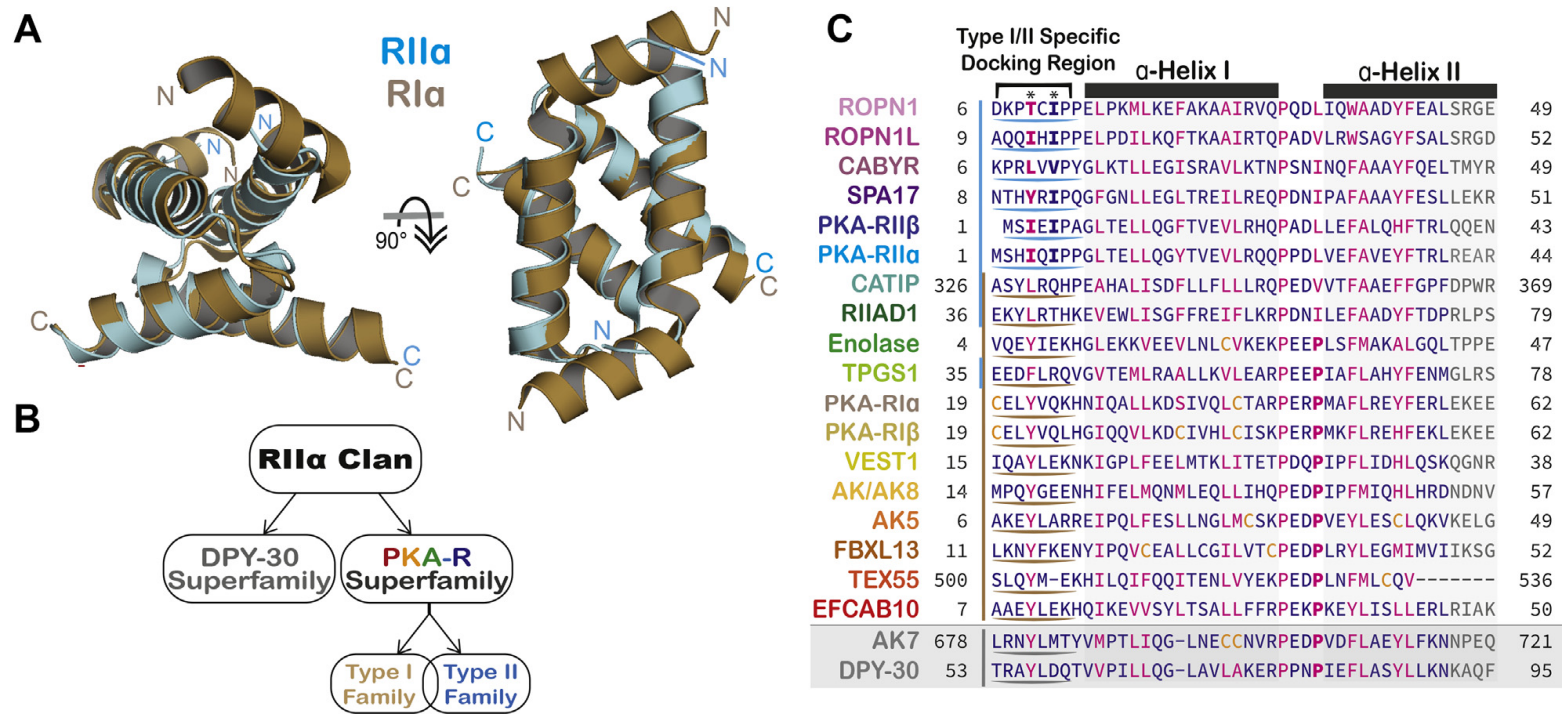
**The RIIα clan and PKA-R D/D domain superfamily.***A*, the RIα (*gold*) and RIIα (*cyan*) D/D domain structures are superimposed. The RMSD over all Cα atoms is 1.406 Å. The *arrow* indicates a 90-degree rotation. *B*, schematic representation of the RIIα clan relationship hierarchy. DPY-30 and PKA-R superfamilies are indicated. The PKA-R superfamily segregates into type I and type II families. *C*, alignment between human PKA-R-like (*multiple colors*) and DPY-30-like (*gray*) D/D domains. Enolase is from the green algae, *Micromonas commoda*. Highly conserved (*magenta*), moderately conserved (*blue*), and nonconserved positions (*gray*) are indicated. Cysteines (*yellow*) are specified. The *bracket* denotes the amino-terminal motif that delineates between type I (*gold*) and type II (*cyan*) D/D domains. Corresponding regions in DPY-30 orthologs (*gray*) are indicated. Positions essential for AKAP interaction are *bolded* and *asterisked*. AKAP, A-kinase-anchoring protein; D/D, docking and dimerization; DPY-30, dumpy-30; PKA-R, PKA regulatory.

While docking grooves were initially believed to be less modular than peptide motifs, the D/D domain is now designated as a *bona fide* modular unit (26, 27, 28). Studies on spermatozoa revealed that anchoring disruptor peptides, such as Ht31, impair flagellar motility (29). Flagellar motility was unaffected by the kinase inhibitor PKI, or the drug H-89 (29). This allowed these authors to conclude that anchored PKA was not involved in this process. Since then, D/D proteins such as sperm autoantigenic protein 17 (SPA17), ROPN1, ropporin-1-like protein (ROPN1L), and CABYR have been recognized as nonkinase AKAP helix–binding partners (28, 30, 31). Functional studies report that genetic ablation of these D/D proteins or loss of association with AKAPs impairs motile ciliary action or flagellar motility (32, 33).

The bioinformatic and phylogenetic studies reported herein classifies the D/D domain superfamily. Eighteen superfamily members are subdivided into type I (R1D2) and type II (R2D2) lineages. Many of these proteins are ancient and present in diverse eukaryotic kingdoms. Others result from a gene expansion that took place at the advent of metazoan multicellularity. To gain further insight into the R2D2 lineage, we determined the crystal structure of apo SPA17 1 to 75 from *Danio rerio*. SPA17 can form homotetramers and displays a low affinity for AKAPs. AKAP binding is considerably enhanced when SPA17 heterodimerizes with another R2D2 protein ROPN1L. Thus, cross-member heterodimerization expands the repertoire and functionality of D/D domains.

## Results

### Annotation of the D/D domain superfamily

A combined strategy for data mining was utilized to generate an improved inventory and annotation of D/D domain–containing proteins. Three databases were interrogated to define relationship hierarchies (Fig. 1*B*). First, the NCBI Conserved Domain Database was searched for proteins within the “Dimerization/docking domain of the regulatory subunit of cAMP-dependent kinase and similar domains”. Second, the SuperFamily library was searched for proteins with the “dimerization-anchoring domain of cAMP-dependent PK regulatory subunit.” Third, protein BLAST analyses against metazoan and excluding metazoan species generated a comprehensive list of the RIIα clan across all taxa. Screening of the P*fam* database refined the RIIα clan as consisting of Dumpy-30 (DPY-30) and PKA-R subunit superfamilies.

The output of our data mining strategy is diagrammatically presented in Figure 1*B*. We defined the RIIα clan as the group comprising the DPY-30 and PKA-R superfamilies. This has led to the identification of 18 PKA-R superfamily members based on sequence identity (Fig. 1*C* and Table 1). The group is further subdivided into type I and type II PKA-R subunit-like D/D proteins (Fig. 1, *B* and *C*).

**Table 1:** Human D/D domain of PKA-R superfamily members

PKA-R DD superfamily	Chr	AA	Mouse KO phenotype
Ropporin-1 (ROPN1)	3	73	♂Subfertility
Ropporin-1-Like (ROPN1L)	5	73	Ciliary dysmotility, ♂subfertility
Ropporin-1B (ROPN1B)	3	73	Not applicable
Ca^2+^-binding Tyr-phosphorylation regulated (CABYR)	18	75	Fibrous sheath dysplasia, ♂subfertility
Sperm autoantigenic protein 17 (SPA17)	11	75	Not available
PKA type II regulatory subunit α (PKA-RII)	3	45	Reduced interaction with AKAPs
PKA type II regulatory subunit β (PKA-RII)	7	45	↑ Metabolic rate (RIIβ) ↓ Body weight/fat (RIIβ)
Ciliogenesis-associated TTC17-interacting protein (CATIP)	2	60	♂Infertility
RIIα domain containing protein 1 (RIIAD1)	1	85	Absent whiskers, abnormal body wall, neonatal lethality
Tubulin polyglutamylase subunit 1 (TPGS1)	19	45	♂Infertility, ↓ body fat, teratozoospermia
PKA type I regulatory subunit α (PKA-RI)	17	50	Carney complex (RIα)
PKA type I regulatory subunit β (PKA-RI)	7	50	↓ LTD and ↓ LTP (RIβ)
Vestibule-1 (VEST1)	8	60	Not available
Adenylate kinase 8 (AK8)	9	60	Hydrocephaly
Adenylate kinase 5 (AK5)	1	65	Not available
F-Box and Leu-rich repeat protein 13 (FBXL13)	7	70	Abnormal eye interior Chamber depth
Testis expressed 55 (TEX55)	3	37	Not available
EF-Hand Ca^2+^ binding Protein 10 (EFCAB10)	7	60	Not available

The gene name of each PKA-R superfamily member as listed in Figure 1*C*. The chromosomal location (Chr) and number of amino acids (AA) are indicated. Putative functions of each family member are inferred by listing the mouse KO phenotype obtained from the Mouse Genomics Data consortium. Not available denotes that a KO mouse has not been generated.

Abbreviations: LTP, long-term potentiation; LTD, long-term depression.

### Taxonomic distribution and phylogeny of the D/D domain superfamily

**Figure 2:**
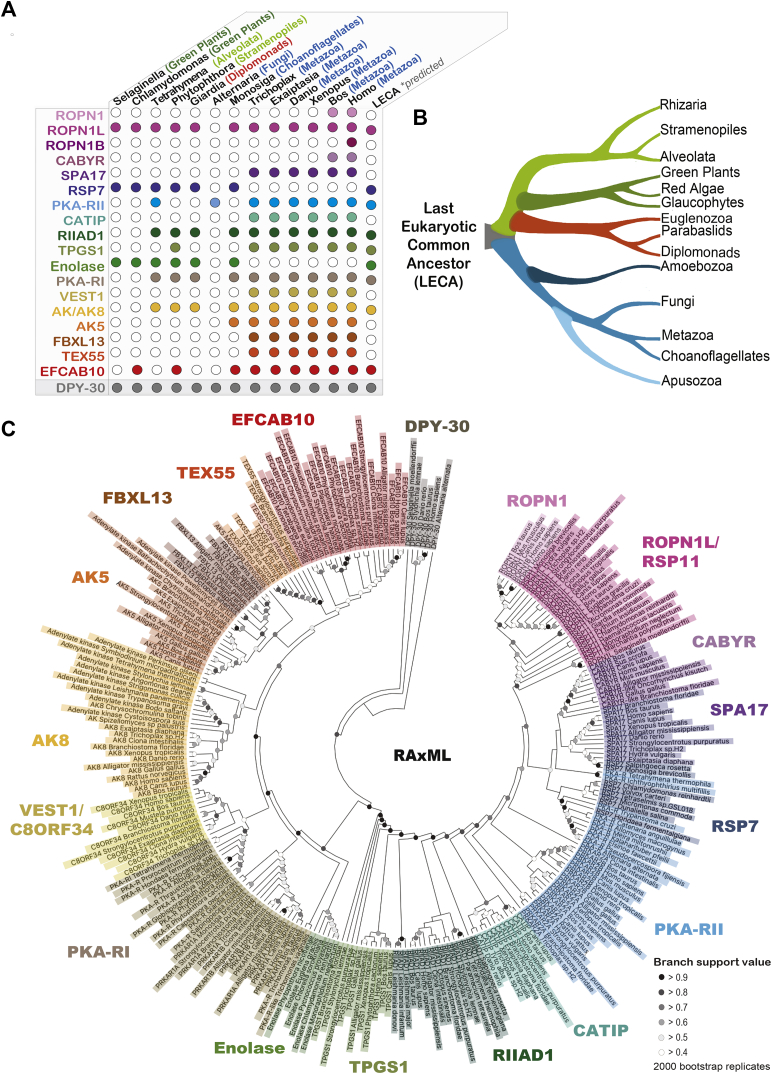
**Taxonomic distribution and phylogenetic topology of the PKA-R D/D domain superfamily.***A*, the table depicting evolutionary conservation of the PKA-R D/D domain superfamily. Each *filled dot* represents the presence of the D/D domain protein identified on the *left* in the genus listed on the *top*. *B*, evolutionary tree illustrates the prevalence of D/D domains across all major eukaryotic clades. *C*, dendrogram displaying the phylogenetic topology of the PKA-R D/D superfamily. 249 orthologs were used to build an estimated phylogeny by maximum likelihood with the RAxML algorithm. The dendrogram was drawn using MEGA-X and represents 2000 combined pseudoreplicates from runs with and without DPY-30 as a designated outgroup. Branch support values are indicated. D/D, docking and dimerization; PKA-R, PKA regulatory.

A total of 249 D/D domain–containing proteins across all taxa were selected for further analysis (Supplemental material). Metazoans have a full complement of PKA-R-like proteins (Fig. 2*A*). These include SPA17, ROPN1L, RIIAD1, CATIP, EFCAB10, TPGS1, AK5, AK8, VEST1, FBXL13, and TEX55. Taxa outside the metazoan kingdom contain the PKA-R-like proteins ROPN1L/RSP11, RSP7, TPGS1, EFCAB10, AK8, enolase, and RIIAD1 (Fig. 2*A*). Higher animals, including humans, additionally evolved the sperm fibrous sheath R2D2 proteins CABYR, ROPN1, and ROPN1B (Fig. 2*A*). The full gene name of each PKA-R superfamily member is listed in Table 1.

An evolutionary tree illustrates how D/D domains evolved across major eukaryotic clades (Fig. 2*B*). Metazoans lost the D/D domain on enolase despite the expansion of the domain to other proteins (Fig. 2, *A* and *B*; Fig. S1). Dendrograms displaying the phylogenetic topology of the D/D superfamily were generated using the RAxML and IQ-tree platforms. Virtually identical branch alignments were obtained on both platforms (Fig. 2*C*; Fig. S1). Interestingly, organisms which do not rely on flagella for reproduction experienced an evolutionary loss of PKA-R-like D/D proteins. For example, gymnosperms and angiosperms use pollen to produce fertile seeds and do not have R2D2 proteins, but mosses and ferns which utilize sperm have R2D2 proteins (Fig. S2).

As previously mentioned, the P*fam* algorithm assigns the DPY-30 and related proteins to the RIIα clan. Our analyses designate the DPY-30 clade as an outgroup that is equally related to RI and RII (Figs. 1*C* and 2*C*). The crystal structure of DPY-30 reveals a D/D fold similar to PKA-R domains (16, 34). Accordingly, the DPY-30 structure is superimposable over RII with an RMSD of 2.6 Å (Fig. S3*A*). Further delineation between DPY-30 and RII is evident from probabilistic hidden Markov modeling (Fig. S3*B*). This algorithm predicts that evolutionary changes have occurred at different positions within the D/D domains of both superfamilies.

Sequence determinants that delineate the RI and RII families predominantly lie in the amino-terminal flanking region and in the loop between helix I and helix II of the D/D domain (Fig. 1*A*). Phylogenetic and topological analyses have used this information to subdivide the PKA-R superfamily D/D domains into two distinct but overlapping groups (Fig. 1, *B* and *C*). A hallmark of RI subunits is the presence of two prolines on each end of the loop between helix I and helix II. Hence, enolase, EFCAB10, AK5, AK8, VEST1, FBXL13, and TEX55 are prototypic of the R1D2 clade (Fig. 1*C*, gold underlined). In contrast, a defining feature of the R2D2 clade is replacement of the second proline with a hydrophobic side chain (Ile, Leu, or Val, Fig. 1*C*, blue underlined). Five proteins, ROPN1, ROPN1L, SPA17, RIIAD1, and CATIP, follow this convention (Figs. 1*C* and 2*C*; Fig. S1).

Other determinants also contribute to the R1D2 or R2D2 designations. For example, TPGS1 is considered an R1D2 protein because it contains a predicted helical flanking amino-terminal motif and a second proline in the loop region. Yet, TPGS1 can also be considered an R2D2 protein because of features such as a glycine at the start of helix I and a conserved “YF” motif in helix II (Figs. 1*C* and 2*C*). Likewise, RIIAD1 and CATIP are intermediate to the R2D2 clade because they are predicted to have an amino-terminal helix rather than a β strand (Fig. 1*C*). Thus, our phylogenetic analyses have defined primary, secondary, and tertiary structure characteristics that are emblematic of the DPY-30, R1D2, and R2D2 subgroups of the RIIα clan. All data have been deposited in the Dryad server.

### Structure of the SPA17 D/D domain

**Figure 3:**
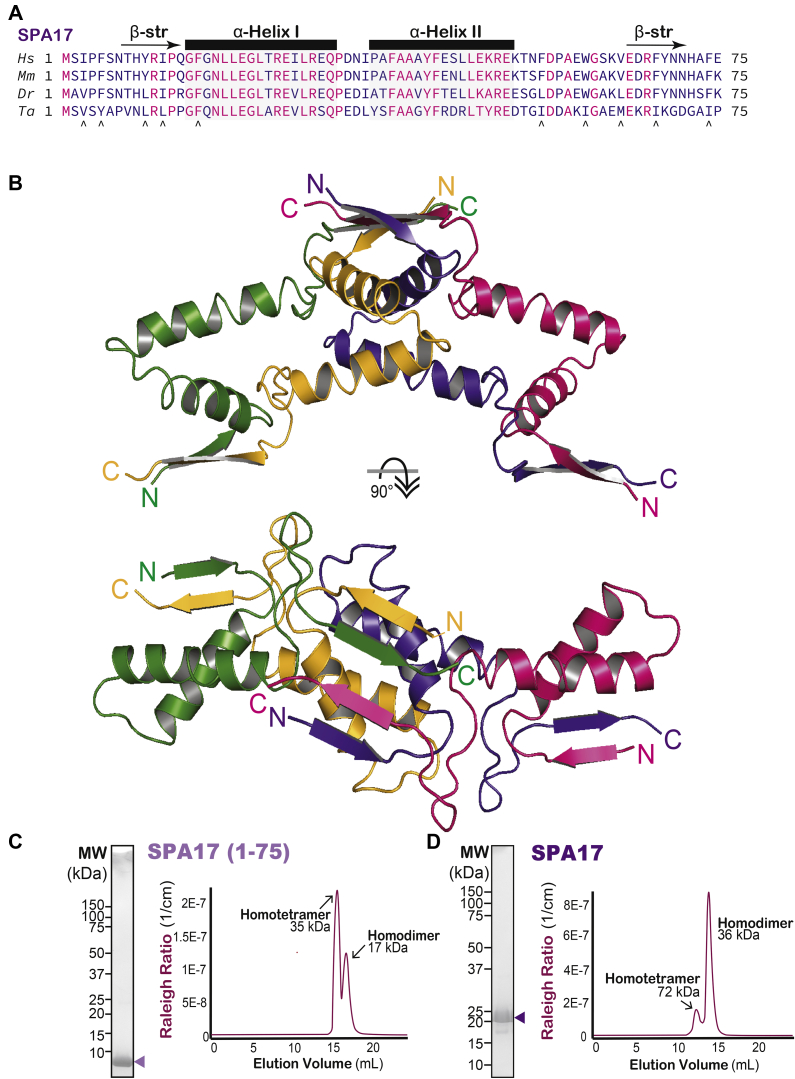
**The structure of SPA17 (1–75).***A*, sequence alignment of human (*Hs*); mouse (*Mm*); zebrafish (*Dr*), and *Trichoplax adhaerens* (*Ta*) SPA17 orthologs. Residues 1 to 75 are presented. *Black arrows* indicate conserved hydrophobic at positions flanking the helical sections. *B*, the first 75 amino acids of SPA17 from *Danio rerio* resolved in a crystal structure at 1.72 Å. *Side view*, four copies of SPA17 (indicated in *green*, *yellow*, *purple*, and *magenta*) are observed in the asymmetric unit of the crystal. *Top view*, extended regions observed in the sequence alignment appear to occlude the AKAP-binding site and facilitate tetramerization. *C* and *D*, the stoichiometry of homomeric SPA17 complexes was calculated by size-exclusion chromatography coupled to multiangle light scattering (SEC-MALS). SDS gels denote protein purity. Molecular weight markers are indicated. *C*, SPA17 1 to 75 with a predicted molecular weight (MW) of 8.8 kDa forms tetramers (35 kDa) and dimers (17 kDa). *D*, full-length SPA17 with a predicted MW of 17.4 kDa forms tetramers (72 kDa) and dimers (36 kDa). Representative gels from three experiments. AKAP, A-kinase-anchoring protein; SPA17, sperm autoantigenic protein 17.

We chose to focus our structural analyses on SPA17 because of its extended D/D domain. The zebrafish ortholog is 72% identical to the human ortholog and proved amenable to crystallization in multispecies trials (Fig. 3*A*). A construct spanning amino acids 1 to 75 of SPA17 from *D. rerio* was expressed in *Escherichia coli*, and the resultant protein was purified with a sequential three-step affinity, anion-exchange, and size-exclusion chromatography (SEC) protocol (Fig. S4). Crystals of SPA17 diffracted X-ray to 1.72 Å. The structure was determined by molecular replacement using the D/D domain of PKA-RIIα as a search model (PDB ID: 2IZX) and subsequently refined to an *R*_*work*_ of 0.154 and *R*_*free*_ of 0.165 (Fig. 3*B* and Table 2).

**Table 2:** Crystallographic data and refinement statistics

Property	Value
Space group	P 32
Cell constants a, b, c, α, β, γ	60.96 Å 60.96 Å 89.02 Å 90.00° 90.00° 120.00°
Resolution (Å)	34.03–1.72 45.41–1.72
% Data completeness (in resolution range)	98.5 (45.41–1.72)
Wavelength	0.99996 Å
Rmeas	0.098
Rpim	0.035
Rmerge (1.72–1.75 Å)	0.91 (0.269)
Data redundancy (1.72–1.75 Å)	7.6 (5.6)
CC1/2	0.993
< I/σ(I) >	1.02 (at 1.72 Å)
Refinement program	phenix.refine 1.18.2_3874, PHENIX 1.18.2_3874
R, Rfree	0.154, 0.165 0.154, 0.166
RMS (angles), RMS (bonds)	0.92, 0.008
Ramachandran favored	100%
Rfree test set	1995 reflections (5.11%)
Wilson B-factor (Å^2^)	11.8
Anisotropy	0.632
Bulk solvent ksol(e/Å^3^), Bsol(Å^2^)	0.42, 34.0
L-test for twinning	< |L| > = 0.51, < L^2^ > = 0.34
Estimated twinning fraction	0.479 for -h,-k,l 0.480 for h,-h-k,-l 0.479 for -k,-h,-l
Fo,Fc correlation	0.96
Total number of atoms	4799
Average B, all atoms (Å^2^)	17.0

Four copies of SPA17 are observed in the asymmetric unit of the crystal. The two central copies of SPA17 form the canonical four-helix bundle as previously observed in the homodimeric D/D domains of R1 and RII (Figs. 1*A* and 3*B*). The other two copies each form a similar homodimer with a symmetry related SPA17 chain. Interestingly, the two conserved sequence regions flanking the central helices of SPA17 both adopt a regular β-strand conformation. A four-stranded β-sheet is formed from the amino-terminal β-strand of two SPA17 molecules and the carboxyl-terminal β-strands of two other SPA17 chains (Fig. 3*B*). Because of the close involvement of these β-strands in crystal packing, the formation of the four-stranded β-sheet is likely a crystallization artifact. Nonetheless, these β-strands might mediate SPA17 oligomerization, as biochemical studies indicate that SPA17 can exist in higher order configurations (Fig. 3, *C* and *D*). SEC coupled to multiangle light scattering (SEC-MALS) verifies that SPA17 tetramers and dimers exist in solution (Fig. 3, *C* and *D*). The SPA17 1 to 75 fragment (8.8-kDa monomer) has molecular masses of 35 and 17 kDa (Fig. 3*C*). Although less evident, multimerization of full-length SPA17 (17.4-kDa monomer) was also observed. The purified protein ensemble elutes with molecular masses of 72 and 36 kDa (Fig. 3*D*). Collectively, the data in Figure 3 imply that, unlike RIIα, SPA17 can form higher order homo-oligomeric complexes.

**Figure 4:**
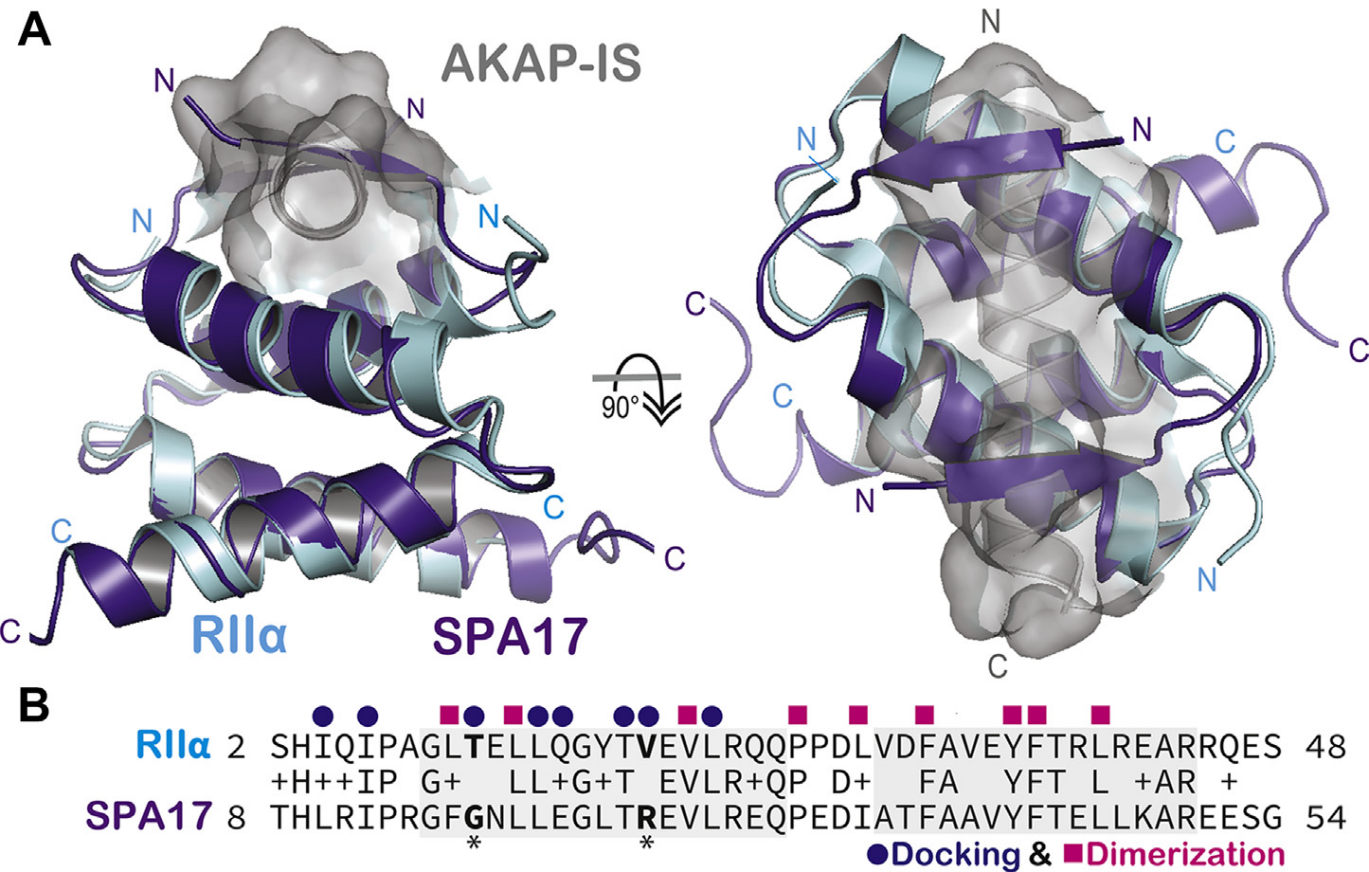
**Comparison of RIIα–AKAP-*IS* interface and Apo SPA17.***A*, structural alignment of murine RIIα D/D domain (*cyan*) in complex with AKAP-*IS* (*gray*, space filling model; PDB ID: 2IZX) and zebrafish SPA17 1 to 75 (*purple*). Alignment with an RMSD of 0.514 Å. *Side view*, extended amino-terminal strands of SPA17 obscure the AKAP-binding site. *Top view*, SPA17 β strands form antiparallel barriers across the AKAP-binding pocket. *B*, sequence alignment of murine RIIα and zebrafish SPA17. Conserved docking (*purple circles*) and dimerization (*magenta squares*) determinants are highlighted. *Bolded* and *asterisked* residues indicate AKAP-docking determinants that differ between RIIα and SPA17. AKAP, A-kinase-anchoring protein; AKAP-*IS*, AKAP-*in silico*; SPA17, sperm autoantigenic protein 17.

Although SPA17 exhibits distinctive structural features, it still retains many hallmarks of a canonical R2D2 protein. Alignment of the SPA17 structure to the D/D domain of apo RIIα dimers results in an associated RMSD of 0.482 Å. Similarly, the apo structure of SPA17 superimposes over RIIα in complex with AKAP-*in silico* with an RMSD of 0.514 Å (Fig. 4*A*, left panel). The AKAP-binding site is, therefore, retained in the SPA17 homodimer, although it appears to be occluded by the β-sheet formed among the terminal strands of SPA17 protomers in the crystal (Fig. 4*A*, right panel). As expected, key hydrophobic residues necessary for dimerization (magenta squares) are strictly conserved, but only five of the docking determinants (purple dots) are invariant in the sequence alignment (Fig. 4*B*). Together, these features suggest that the extended D/D domain of SPA17 contains most necessary determinants for binding to AKAPs. Thus, the mode of SPA17 interaction with AKAPs might be slightly different than how RII interfaces with its anchoring proteins.

### SPA17–ROPN1L heterodimers form a functional AKAP-binding module

**Figure 5:**
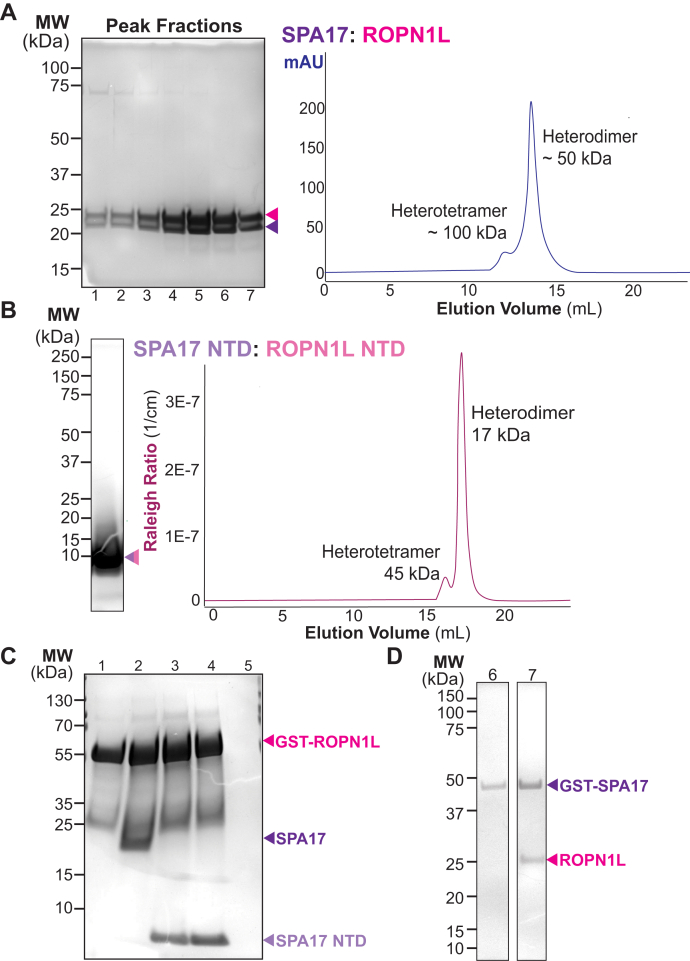
**SPA17 heterodimerizes with ROPN1L.***A*, size-exclusion chromatography evaluated complex formation between SPA17 and ROPN1L. *Left panel*, Coomassie-stained SDS gel of peak fractions (indicted below each lane). *Right trace*, SPA17–ROPN1L multimerization assayed by SEC. Apparent molecular weights of heterotetramer and heterodimer are indicated. *B*, size-exclusion chromatography coupled to multiangle light scattering (SEC-MALS) evaluated SPA17 1 to 75 complex formation with ROPN1L 1 to 75. *Left panel*, Coomassie-stained SDS gel of peak SPA17 1 to 75–ROPN1L 1 to 75 fraction. *Right trace*, SPA17: ROPN1L multimerization assayed by SEC-MALS. Apparent molecular weights of heterotetramer and heterodimer are indicated. *C* and *D*, reciprocal GST pull-down experiments demonstrate interaction between GST-ROPN1L (*pink*) and SPA17 (*purple*) and SPA17 1 to 75 (*lavender*). *C*, Coomassie-stained SDS gel shows GST-ROPN1L (lane 1). Pull down of full-length SPA17 (lane 2), human SPA17 1 to 75 (lane 3), zebrafish SPA17 1 to 75 (lane 4), and beads only (lane 5). *D*, Coomassie-stained SDS gel of reciprocal GST-pull-down binding experiments. Protein GST-SPA17 (lane 1). ROPN1L binding to GST-SPA17 (lane 2). Molecular weight markers are indicated. Representative gels of three experiments. SPA17, sperm autoantigenic protein 17.

SPA17 coexists with ropporin-1-like proteins in the flagellum of mammalian sperm and motile cilia (31). Sequence similarities between these members of the R2D2 clade raised the possibility that SPA17 and its close relative ROPN1L may form heterodimers (Fig. 1*C*). In keeping with this notion, full-length SPA17 and ROPN1L comigrate as assessed by SEC-MALS analysis (Fig. 5*A*). Likewise, SPA17 1 to 75 and ROPN1L 1 to 75 multimerize when analyzed by SEC-MALS (Fig. 5*B*). Gel filtration traces further indicate that SPA17–ROPN1L complexes migrate with an apparent molecular weight that is consistent with a heterodimer with a minor tetrameric species (Fig. 5, *A* and *B*). Protein pulldowns verified interaction between glutathione-*S*-transferase (GST)-ROPN1L and SPA17 (Fig. 5*C*). Reciprocal pull-down experiments confirmed that GST-SPA17 binds ROPN1L (Fig. 5*D*). Thus, SPA17 has the capacity to form heterodimers with ROPN1L.

**Figure 6:**
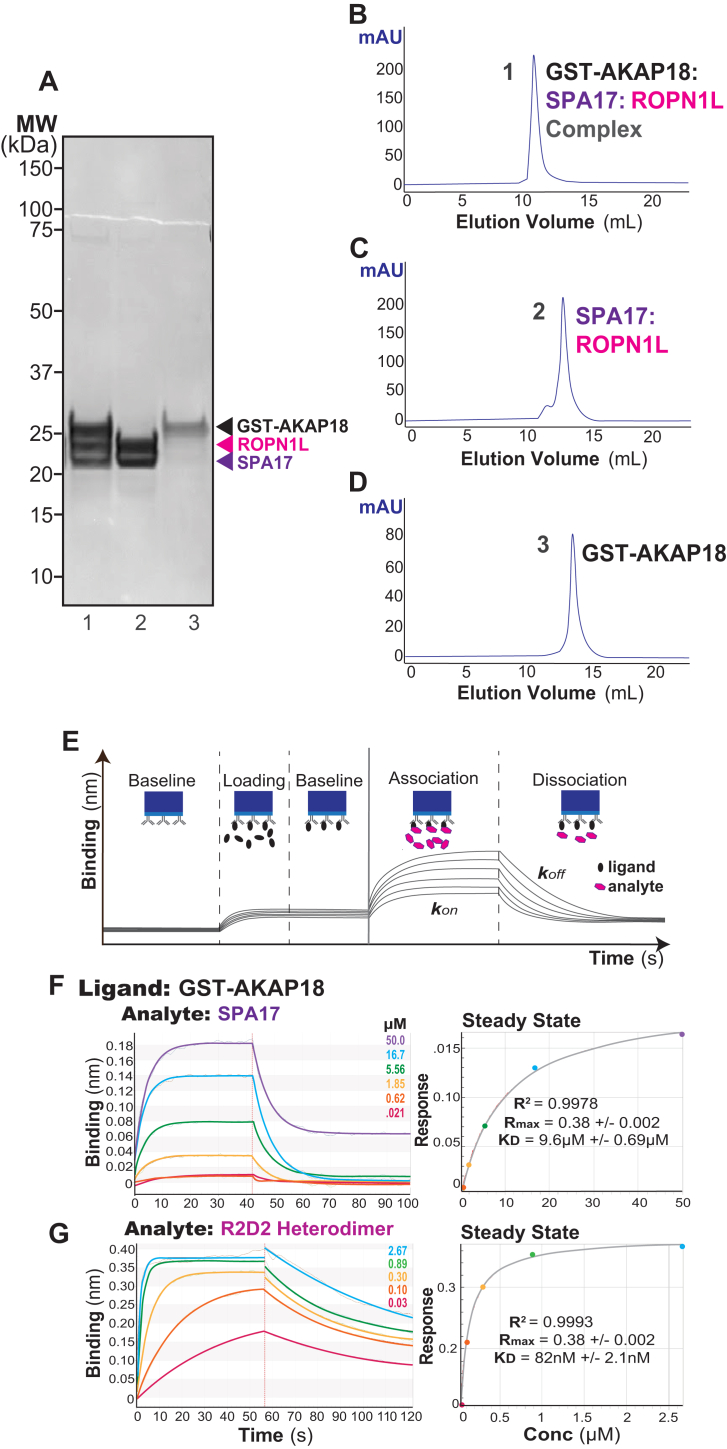
**The SPA17–ROPN1L heterodimer is the functional AKAP-binding module.***A*, Coomassie-stained gel showing the peak fractions from the gel filtration experiment to the right. Electrophoretic mobility of GST-AKAP18 (*gray*), ROPN1L (*magenta*), and SPA17 (*purple*) and molecular weight markers are indicated. *B*, GST–AKAP18–SPA17–ROPN1L ternary complex (lane 1 in panel *A*) elutes in a single peak in 10.4 ml. *C*, SPA17–ROPN1L heterodimer (lane 2 in panel *A*) elutes in 13.1 ml. *D*, GST-AKAP18 (lane 3 in panel *A*) elutes in 13.4 ml. *E*, diagram of the Octet BLI system. In the first step, a probe with anti-GST antibodies is washed with a buffer. GST protein is applied to the probe, and the detector shows a shift in the refractive index corresponding to increased mass on the surface of the probe. Excess protein is washed away with the buffer to record a baseline. Finally, the ligand of interest is loaded and washed to calculate the binding kinetics. Each color trace represents an increasing concentration of the ligand (as indicated). *F*, SPA17 alone (*top subpanel*) has micromolar affinity for AKAP18. *G*, SPA17 1 to 75–ROPN1L 1 to 75 heterodimer has nanomolar affinity for AKAP18. *F* and *G*, *left subpanels* show raw data with *fitted curves* to calculate *kon* and *koff*. *Right subpanel* graphs are calculated as follows: $Response\;=\left(R_{max}\times \;concentration\right)÷\left(K_{D}+\;concentration\right)$. Amalgamated data from five experiments derived dissociation constants. AKAP, A-kinase-anchoring protein; BLI, biolayer interferometry analysis; SPA17, sperm autoantigenic protein 17.

Homomeric SPA17 exhibits limited interaction with AKAPs (31). However, AKAP interaction is more evident in cells that co-express SPA17 and ROPN1L (31). Therefore, it was imperative that we investigated the AKAP-binding properties of SPA17–ROPN1L heterodimers. SEC-MALS was used to evaluate full-length SPA17 and ROPN1L interaction with a GST-AKAP18 fusion (35). In solution, the AKAP18–SPA17–ROPN1L ternary complex elutes as a single peak at 10.4 ml that corresponds to a molecular weight ∼330 kDa (Fig. 6*A*: lanes 1 and Fig. 6*B*). This is equivalent to a tetrameric GST-AKAP18–SPA17–ROPN1L assembly. In contrast, SPA17–ROPN1L heterodimers (Fig. 6*C*) and GST-AKAP18 (Fig. 6*D*) elute with lower apparent molecular weights on the analytical size-exclusion column (Fig. 6*A*: lanes 2 and 3).

To quantify the AKAP18 interaction with SPA17 and ROPN1L, biolayer interferometry analysis was performed on the Octet system. A probe with anti-GST antibodies was loaded with GST-AKAP18, washed, and then incubated with either SPA17 alone or SPA17–ROPN1L over a range of concentrations. The steady state affinity is calculated as $Response\;=\left(R_{max}\times \;concentration\right)÷\left(K_{D}+\;concentration\right)$. Homomeric SPA17 bound AKAP18 with an affinity of 9.6 ± 0.69 μM (Fig. 6*F*), supporting the notion that its AKAP-binding site is not blocked by the terminal β strands. Remarkably, the SPA17–ROPN1L heterodimer binds AKAP18 with an affinity of 82 ± 2.1 nM (Fig. 6*G*). For comparison, the *K*_*d*_ for RIIα interaction with AKAP18 is 31 nM (25). Hence, SPA17–ROPN1L heterodimers exhibit a physiologically relevant binding affinity for AKAP18 and are likely to represent the preferred AKAP-binding module. To further probe this phenomenon, we conducted *in silico* modeling of the AKAP18–SPA17–ROPN1L trimer (Fig. S5, *A* and *B*). Sequence alignments indicate that the AKAP18-anchoring helix is palindromic except for the central two residues (Fig. S5, *C* and *D*). Hence, the AKAP helix can be presented in two possible orientations (Fig. S5, *A* and *B*). Several factors could favor one of the orientations and lead to an enhanced affinity in comparison with the SPA17 homodimer. These include the asymmetric nature of the SPA17–ROPN1L complex, the distinct pI values of both protomers and differential exposure of core DD domain residues. In addition, the unique N-terminal flanking sequences on SPA17 and ROPN1L might participate in customized protein–protein interactions that enhance affinity for the AKAP18 helix only in the context of the heterodimer.

## Discussion

Our studies shed new light on the evolution of D/D domains. These regions were originally characterized as amino-terminal elements of the regulatory subunits of PKA that form homodimers to create an AKAP-binding surface (13, 16). Our subsequent identification of 18 family members presented in Figures 1 and 2 suggests that these four-helix bundle-like–forming proteins have a broader role in shaping subcellular architecture than previously appreciated. Another interesting outcome of our phylogenetic analysis has been the segregation of the D/D domain into the DPY-30 and the PKA-R superfamilies. DPY-30 proteins are universally found in all phyla and may be more closely related to the primordial D/D domain. In contrast, there are signature motifs within the PKA-R superfamily that subdivide this group into the R1D2 and R2D2 families. Each D/D domain class exhibits distinct features that contribute to their specialized roles in the coordination of organellar and subnuclear events.

R1D2 proteins are typified by a flanking N-terminal helix containing consecutive aromatic and hydrophobic amino acids and a loop with a PxxP motif. Conversely, R2D2 proteins have a β strand and a Pxx[L,I,V] motif in the corresponding regions. Our phylogenetic analyses in Figures 1*C* and 2*A* indicate that there was an expansion in both classes around the time of metazoan evolution. In contrast, fungi and plants have fewer D/D proteins. For example, *BCY1* the PKA-R subunit gene in the budding yeast *Saccharomyces cerevisiae* contains a recognizable RII-like D/D domain that also forms crystallization-related oligomers (36, 37, 38, 39, 40). Structural evidence for the oligomerization of *BCY1* calls into question the ability of this D/D domain to dock with fungal proteins (36). Parenthetically, there is scant evidence for AKAPs in the fungi kingdom (2, 3, 4). This may be because kinase anchoring is not a preferred mechanism for local signaling in these less morphologically developed organisms, and subcellular organelles including cilia are not prominent in these kingdoms. Taken together, this data mining venture indicates that metazoans express the most comprehensive set of D/D domain proteins, thereby lending further credence to the notion that compartmentalized signaling expanded at the base of animal evolution.

DPY-30 proteins are an outgroup to the D/D family that are present in all animal and plant phyla (41). The founding member DPY-30 encodes the core subunit of the SET1/MLL family of COMPASS histone methyltransferases (42). DPY-30 homodimers associate with a helical region on ASH2L, another core element of the COMPASS complex to stabilize intrinsic methyltransferase activity (43, 44). This protein–protein interaction bears a striking resemblance to the RI–AKAP and RII–AKAP interfaces as depicted in Fig. S6, *A* and *B*. In agreement with this concept, the DPY-30 four-helix bundle retains the capacity to interact with AKAPs and is thought to be a native ligand for AKAP95. This affords a means to incorporate AKAP95 into histone methyltransferase complexes (45). Previous studies have shown that AKAP95 is a predominantly nuclear protein that has the capacity to interact with PKA but only when the nuclear envelope is dissolved during mitosis (46, 47). Nonetheless, AKAP95–PKA holoenzyme complexes may participate in different signaling events during cell division, as a significant proportion of the anchoring protein remains associated with DPY-30 during mitosis (45, 48). Thus, differential association with RII or DPY-30 may determine whether AKAP95 functions as a kinase-anchoring protein or an ancillary component of histone methyltransferases. The latter function may also be relevant to AKAP95 nuclear role in interphase cells as this anchoring protein has been implicated as a positive regulator of pre-mRNA splicing and gene expression during tumorigenesis (49, 50). Overall, these observations imply that subtle but conserved differences in the D/D domain not only influence which binding partners this protein module interacts with but also have marked effects on the differential functionality of the resulting macromolecular complexes.

Although SPA17 and RIIα are homologous, our sequence analysis identified unique residues in the helical flanking regions of SPA17. For this reason, we determined the crystal structure of the extended D/D domain of SPA17. Interestingly, sequences at either end of the helical segments form a four-stranded β sheet that occludes the AKAP interface. Although AKAP-binding determinants are conserved in the core of SPA17, our *in vitro*–binding studies presented in Figure 6 reveal that the anchoring protein AKAP18 has a surprisingly low affinity toward this homomeric D/D domain. Although interference from flanking regions could explain poor AKAP binding, it is currently unclear whether or not this molecular mechanism is operational in the context of the native protein. Likewise, participation of these flanking β strands in the crystal packing that lead to the formation of a tetramers could be construed as a consequence of the unnatural physiochemical conditions imposed by protein crystallization. Yet, our SEC and MALS experiments presented in Figure 3, *C* and *D* indicate that soluble SPA17 exists in both dimeric and tetrameric configurations. Thus, an equally plausible explanation is that the conserved flanking regions facilitate supplementary oligomerization of SPA17.

Analogous flanking regions are detected in other R2D2 family members. In addition, proteomic screens often report that clusters of D/D domain proteins exist within the same macromolecular complexes (51). This raises the intriguing possibility that certain D/D domain proteins have the capacity to heterodimerize. Consistent with this notion, our biochemical studies presented in Figures 5 and 6 show that SPA17 can dimerize with the ROPN1L. This latter D/D protein also features extended flanking regions that are highly conserved in all orthologs. A particularly fascinating aspect of this observation is that SPA17–ROPN1L heterodimers display a higher affinity for AKAPs as assessed by our quantitative binding measurements presented in Figure 6*G*. Heterodimerization may be a result of the complementary isoelectric point values of ROPN1L and SPA17 N-terminal domains as indicated in Fig. S5*E*. The increased affinity for AKAP binding is likely due to central asymmetric residues in the context of a largely palindromic AKAP helix binding to distinct residues exposed within the binding grove formed by the SPA17–ROPN1L heterodimer. Indirect support for this concept is provided by analysis of the closely related R2D2 proteins, RSP7 and RSP11 (52). In this context, the N-terminal flanking regions are observed to form interactions with distal portions of amphipathic helix on their binding partner RSP3 (as depicted in Fig. S5, *A* and *B*). Thus, the formation of mixed four-helix bundles could expose additional AKAP-binding determinants that are masked in homomeric conformations. Alternatively, flanking regions of the SPA17–ROPN1L heterodimers might not be able to interfere with the AKAP interface. Irrespective of which explanation is correct, our discovery of D/D domain heterodimers expands the repertoire of D/D modules that may be operational within the intracellular environments.

A recurring theme throughout this study is evidence that D/D domain proteins participate in the organization and structural integrity of flagella and motile cilia (51). The rhythmic beating of these motile appendages enhances microorganism motility and the propulsion of sperm. Interestingly, the sperm fibrous sheath and ciliary radial spoke complexes are organized by AKAPs and D/D proteins (53, 54). Moreover, our exploration of D/D heterodimerization presented in Figures 5 and 6 is reminiscent of the recent cryo-EM analyses of the axonemal radial spoke complex (52, 55). The radial spoke is a complex of 23 proteins that functions as a mechanochemical transducer that modulates activity of dynein motors to promote flagellar motility. Eukaryotic flagella and motile cilia share a common “9 + 2” structure, in which nine peripheral microtubule doublets surround the central pair of microtubules. These substructures are connected by radial spokes. The radial spoke is a T-shaped macromolecular assembly that anchors peripheral microtubules to the central pair. The stalk of the radial spoke is organized by an anchoring protein called RSP3 that coordinates microtubule sliding (52). The radial spoke was originally thought to contain PKA by virtue of evidence that RII binds RSP3 in a far-Western overlay assay (56, 57). However, proteomic screens have never detected PKA as a component of this complex (53). Likewise, the *Chlamydomonas reinhardtii* protein RSP3, which originally was designated as an AKAP, is now recognized to anchor RSP7–RSP11 heterodimers. This heterodimeric complex of D/D proteins might provide rigidity to this flagellar substructure (52). The architectural integrity imparted by RSP7–RSP11 heterodimers involves two classes of protein–protein interaction. The D/D domain tethers to RSP3, whereas binding motifs in each protomer cross-link with other elements of the radial spoke. Interestingly, algal RSP11 is an ortholog of metazoan ROPN1L. Likewise, RSP7 and SPA17 share a conserved extended D/D domain and related calcium-binding motifs. In total, these observations infer that this D/D–AKAP interface is an adaptable architectural element, rather than just a platform for kinase signaling.

In conclusion, this study highlights the discovery and classification of interactive motifs patterned after the D/D domains of the PKA-R subunits. We show that this emergent protein module functions as an AKAP-interaction domain. Undoubtedly, the R1D2 and R2D2 proteins described herein create platforms for intracellular signaling. However, these D/D domains may equally serve as architectural components of motile cilia and integration points for processing cellular cues including calcium and histone methylation. Moreover, cross-member heterodimerization may represent a previously unconsidered mechanism to expand the repertoire and functionality of D/D domains well beyond the compartmentalization of PKA. Future studies are necessary to elucidate roles of the RIIα clan as multipurpose adapters that are utilized in expanding number of molecular and cellular contexts.

## Experimental procedures

### Phylogenetic analysis

A combined approach was used to inventory PKA-R-like D/D domain–containing proteins. In an attempt to identify all putative R1D2 and R2D2 proteins, the NCBI Conserved Domain Database was searched for proteins within the “D/D-R-PKA: Dimerization/Docking domain of the Regulatory subunit of cAMP-dependent kinase and similar domains” superfamily and the superfamily Hidden Markov models, and genome assignment library was searched for proteins within the “Dimerization-anchoring domain of cAMP-dependent PK regulatory subunit” designation. The SuperFamily library contained all of the human PKA-R and related proteins. An initial list of species and initial alignment spanning about 45 amino acids was created using the SuperFamily server (58). To obtain the most comprehensive list of taxa possible, NCBI BLAST analysis was conducted against metazoan and excluding metazoan species. A total of 249 proteins were chosen across all taxa for use in further analysis. The metazoan representatives were from cnidarian, placozoan, molluscan, echinoderm, tunicate, lancelet, fish, reptile, bird, and multiple mammalian taxa. An attempt was made to include all nonhypothetical non-opisthokont orthologs to resolve ancient lineages of the PKA-R-like proteins. The phylogenetic bootstrap values may have been higher by only including metazoans, but instead, the most comprehensive rather than the most stable taxa were chosen for an analysis with the highest information content.

An alignment of the 249 protein representatives was conducted in MEGA-X using MUSCLE (59, 60). Preliminary maximum likelihood runs on MEGA-X had low bootstrap support values on the main branches, and the alignments were extended to include the flanking regions to the core helices for a total of 77 amino acids for each protein (where available). These sequences were aligned by both MUSCLE and CLUSTAL-W (61), but the CLUSTAL-W alignment was of higher quality with fewer gaps, so it was chosen for further analysis and uploaded to the CIPRES Science Gateway for the inference of large phylogenetic trees (62).

To determine the optimal parameters for the maximum likelihood run, the data were input and analyzed by JModelTest2 (63) on XSEDE. This program predicted a maximum likelihood analysis with a gamma distribution of four categories plus invariant sites using the LG amino acid substitution matrix would be the highest quality, so these parameters were utilized. Phylogeny estimation by maximum likelihood analysis was conducted using RAxML-NG (64) on XSEDE with 1000 replicate trees for calculation of transfer bootstrap expectation support metrics. This analysis was conducted twice, once with DPY-30 as an outgroup and once without an outgroup. The resulting dendrograms were identical, so the branch support values were listed for 2000 replicate trees. A second maximum likelihood analysis was conducted for comparison using IQ-Tree (65, 66) on XSEDE with 2000 replicates trees for calculation of UFBoot support metrics. Dendrograms were drawn and visualized in MEGA-X.

### Molecular biology and protein purification

Human ROPN1L, ROPN1L (1–75), SPA17, SPA17 NTD (1–75), SPA17 NTD (1–80), AKAP18α, and zebrafish SPA17 (1–75) were cloned into pGEX-6P-1 using the BamHI and XhoI restriction sites. These plasmids were then transformed into BL21 cells from Invitrogen for protein expression. Induction was performed at 16 °C overnight using 1 mM IPTG starting at an absorbance at 600 nm of about 0.8. Cells were lysed in a buffer containing 50 mM Tris, pH 8, 200 mM NaCl, and 5 mM DTT. Lysates were clarified *via* centrifugation at 40,000*g*. The clarified lysate was first purified using a glutathione affinity column (GE Healthcare) pre-equilibrated in the lysis buffer. Protein was eluted from the column by overnight on-column cleavage of the GST tag by 3C-HRV protease produced in house at 4 °C. Eluted material was further purified using the AKTA system with a 5-ml HiTrap Q-HP column (GE Healthcare), followed by a Superdex 200 gel filtration column (GE Healthcare) in a final buffer containing 10 mM Tris, pH 8, 200 mM NaCl, 5 mM DTT (Fig. S5). Proteins were concentrated to 15 to 20 mg/ml, flash-frozen in liquid nitrogen, and stored in −80 °C.

### Crystallization

Extensive crystallization screening was conducted to obtain high-quality crystals. Purified *D. rerio* SPA17 NTD (1–75) protein was mixed with mother liquor in a 1:1 ratio using 150 nl of each component and spotted onto hanging drop seals on a Mosquito nanodrop robot. The crystallization trays were then placed at 4 °C. The crystals used for analysis grew in 0.1 M Hepes, pH 7.5, and 0.5 M magnesium formate dihydrate from the Index Screen by Hampton Research. Crystals appeared after 2 to 3 days and reached their final size in 1 to 2 weeks. Crystals were directly transferred to a cryo-protectant buffer containing 50% glycerol and 50% mother liquor and frozen in liquid nitrogen for synchrotron data collection.

### X-ray data collection and structure determination

X-ray diffraction data were collected at the Advanced Light Source at Lawrence Berkeley National Laboratory. Diffraction data were indexed, integrated, and scaled with the HKL-2000 package (67). Resolution cutoffs were determined using completeness (>80%) and I/σ >1 as primary criteria. Initial phases were determined by the molecular replacement method using PKA RIIα (PDB ID: 2IZX) as a search model. The majority of the model was subsequently build using the autobuild function in PHENIX (68). Minor rebuilding was done manually in Coot (69). Refinement was also conducted in PHENIX, and the final model had an *R*_*work*_*/R*_*free*_ of 0.154/0.165. All structural figures used for data analysis were visualized in PyMOL.

### Protein pull-down

GST-AKAP18α and GST-RIIα were transformed into BL21 cells and purified using glutathione-affinity purification and anion-exchange chromatography. About 0.3 mg of purified GST proteins, GST-AKAP18α, GST-SPA17, GST-ROPN1L, and GST-RIIα was loaded onto a GST purification column (GE Healthcare) in a bed volume of 200 μl. Solutions containing 0.3 mg of SPA17 NTD, SPA17, or ROPN1L were then applied to the column, which was washed three times with ten column volumes of the lysis buffer (50 mM Tris, pH 8, 200 mM NaCl, 5 mM DTT). Samples were eluted with 10 mM glutathione in the lysis buffer, mixed with SDS-PAGE loading buffer, analyzed by SDS-PAGE, and visualized by Coomassie staining.

### Gel filtration chromatography and SEC-MALS analysis

Gel filtration chromatography was performed using an AKTA system with a Superdex 200 Increase 10/300 Gel Filtration column in 20 mM Tris, pH 8, 200 mM NaCl, and 5 mM DTT at a flow rate of 0.5 ml/min. MALS analysis was conducted using a Wyatt system attached to a Superdex 200 Increase 10/300 gel filtration column in the same buffer with the same flow rate.

### Octet biolayer interferometry measurement

Binding affinity was measured using purified GST-AKAP18α on the Octet RED96 (ForteBio, Pall Life Sciences). The reaction was carried out in black 96-well plates. The reaction volume was 200 μl in each well, and the reaction was carried out at room temperature. The optical probes contained anti-GST to capture GST-AKAP18α that was loaded at a concentration of 200 nM. The binding buffer contained 50 mM Tris HCl, 200 mM NaCl, 5 mM DTT, and 0.1% BSA, pH 8.0. Concentrations of 50, 16.7, 5.56, 1.85, 0.617, and 0.2058 μM SPA17 homodimer, and 2.67, 0.889, 0.2963, 0.0988, and 0.329 μM SPA17 NTD ROPN1L NTD heterodimer were loaded onto the probes. The analyte did not bind to the unloaded probes or a probe containing GST-His alone at the same 200 nM concentration. Binding kinetics for all concentrations were measured at the same time using default instrument settings. Data analysis was conducted with Octet data analysis software. The association and dissociation curves were locally fit over the entire step time with a 1:1 ligand model. Steady-state analysis was used to determine the affinity constant *K*_*D*_ from the calculated equilibrium response with the following equation: Response = (Rmax ∗ concentration)/(*K*_*D*_ + concentration).

### Protein quantitation

Protein quantitation was conducted using a NanoDrop with A280 extinction coefficients calculated for each protein using ExPASy ProtParam.

## Data availability

All data are contained within the article. Coordinates for the crystal structure of SPA17 Docking and Dimerization Domain from *Danio rerio* have been deposited in the PDB database. Accession Number: PDB ID: 7MY4.

## Supporting information

This article contains supporting information.

## Conflict of interest

The authors declare that they have no conflicts of interest with the contents of this article.
