# Supplementary material for: Beyond PKA: Evolutionary and structural insights that define a docking and dimerization domain superfamily
Source: J Biol Chem. 2021 Jul 10;297(2):100927. doi: 10.1016/j.jbc.2021.100927 (PMC8339350; doi:10.1016/j.jbc.2021.100927)
Supplement: Supplemental Figures S1–S6 [file mmc1.pdf]

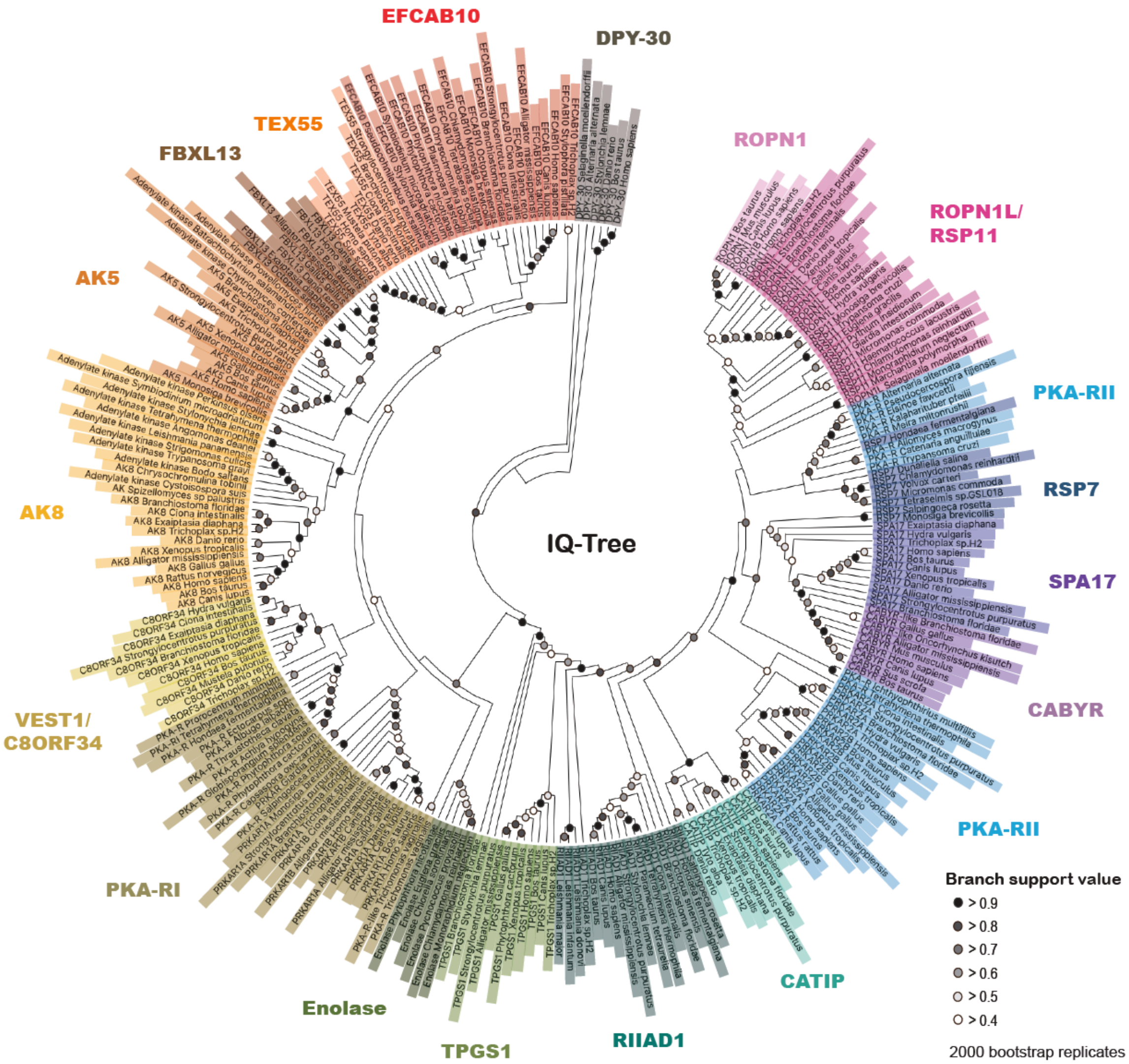

Supplementary Figure S1. Dendrogram of 249 D/D domain orthologs built with an estimated phylogeny by maximum likelihood using the IQ-Tree algorithm. Branch support values are indicated.

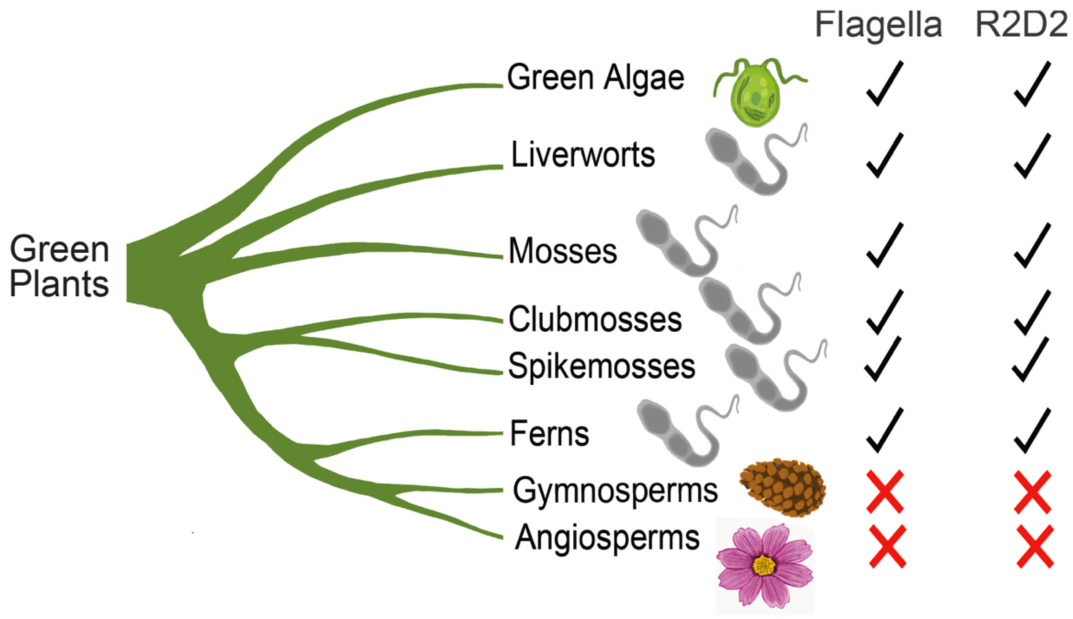

Supplementary Figure S2. Evolutionary tree of green plants reveals loss of R2D2 proteins is coincident with the loss of sperm for reproduction. Check marks indicate the presence of flagella and R2D2 proteins.

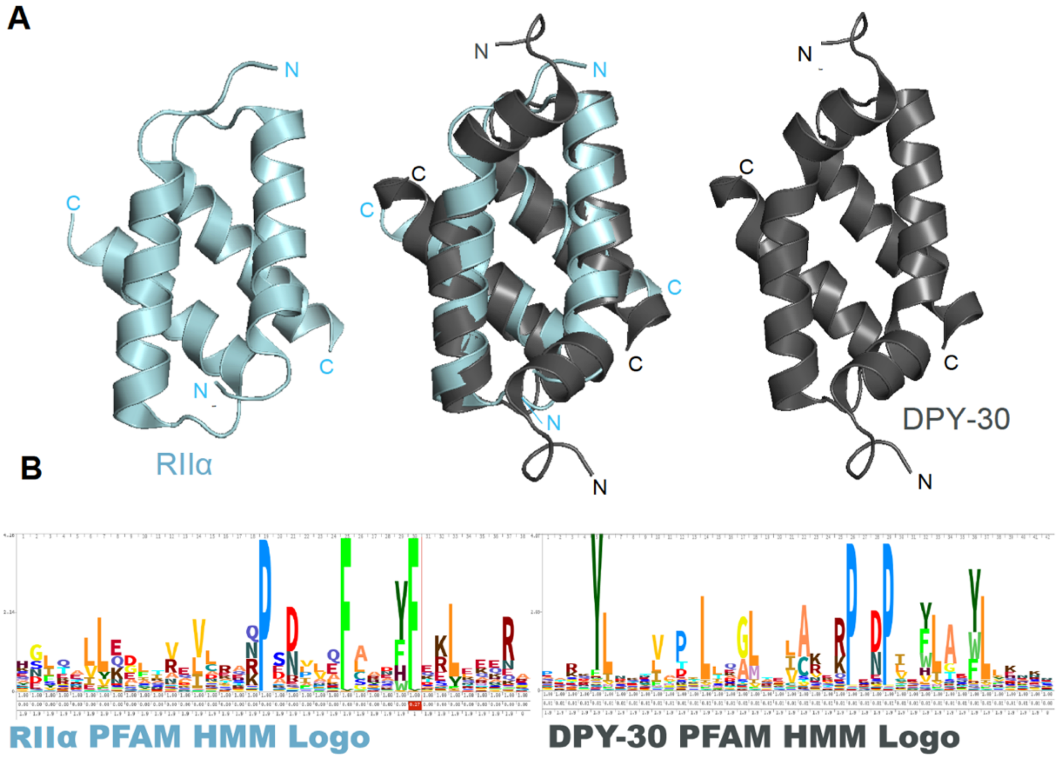

Supplementary Figure S3. A) D/D domain structures of RIIα (cyan) and DPY-30 (charcoal) are superimposed. The root-mean-square deviation (RMSD) is 2.6 Å. N and C termini are indicated. **B)** Probabilistic hidden Markov modeling predicts that evolutionary changes at different positions within both D/D domains. Amino acids are indicated in the one letter code. The size of each letter indicates the probability of that residue occupying this position on the D/D fold.

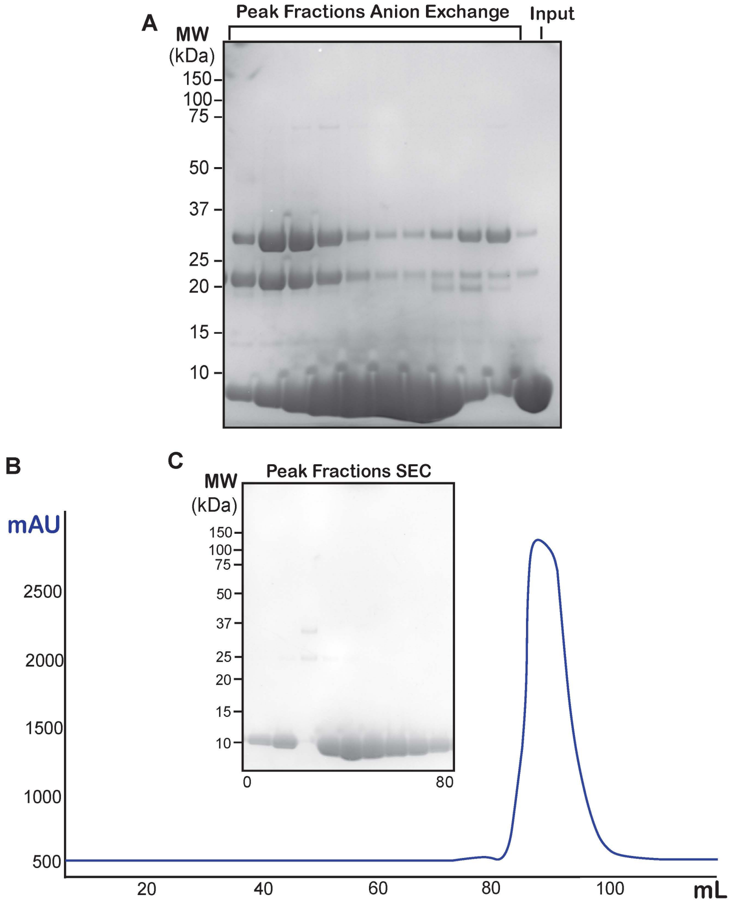

Supplementary Figure S4. Purification of Zebrafish SPA17 1-75. A) Coomassie stained gel of purification by anion exchange and affinity chromatography (last lane: input). **B)** Size exclusion chromatography filtration (SEC) profile using the 120 mL Prep Grade column. Molecular weight markers are indicated. **C)** Coomassie stained gel of purification by size exclusion chromatography.

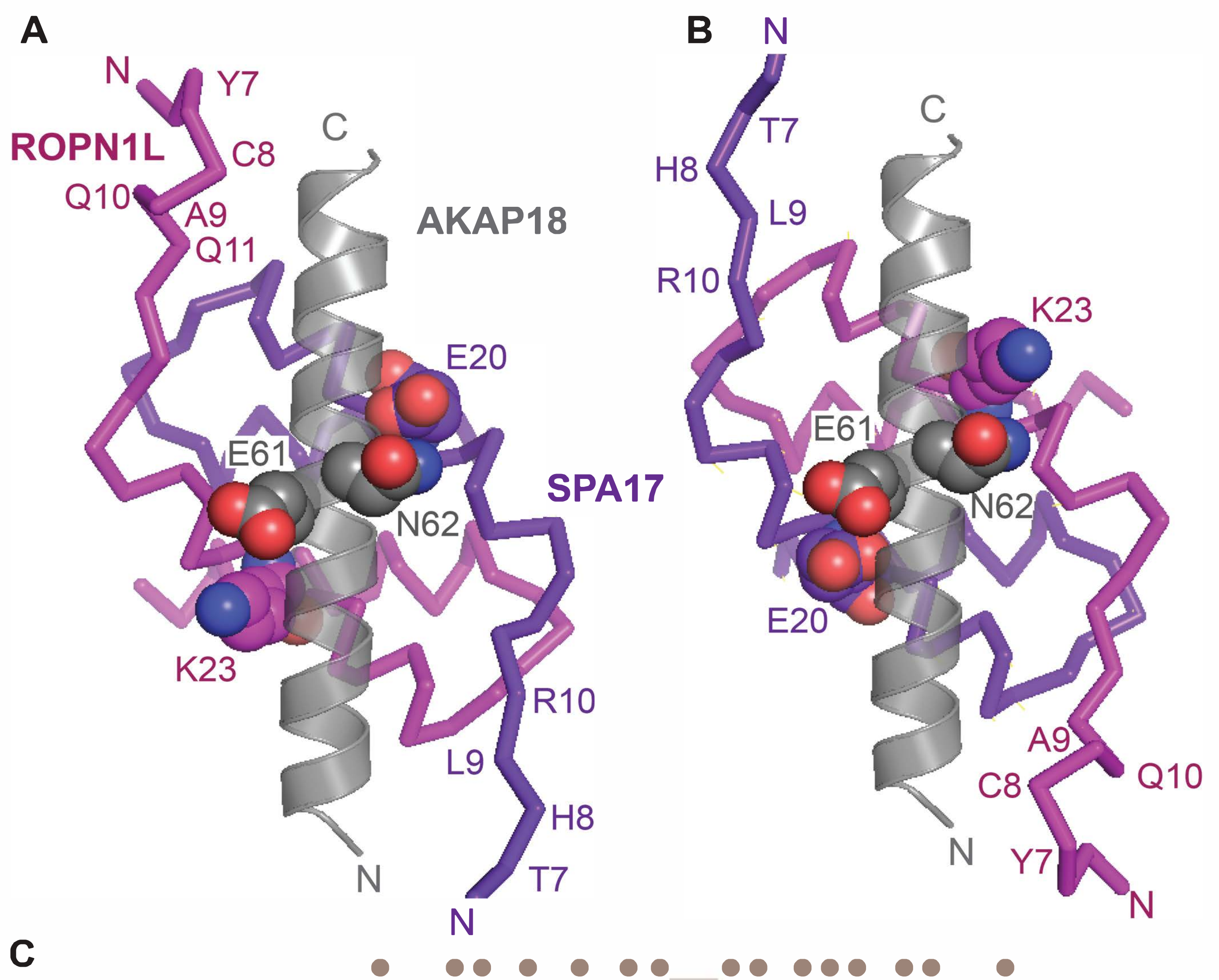

C

```
AKAP18  49 DAELVRLSKRLVENAVLKAVQQYLEET 76
  RSP3 155 DFEVEPILEVLVGKVLEQGLMEVLEEE 180
```

D

```
SPA17  4  FSNTHLRIPRGFGNLLEGLTREVLREQPEDIATFAAV 42
ROPN1L 8  YCAQQINIPPALPNMLKQFTKAAIRTQPRDVLQWAAD 46
```

E

	pI
SPA17 (1-75):	5.3
ROPN1L (1-72):	9.3

Supplementary Figure S5. Modeling of AKAP18-SPA17-ROPN1L trimer. A) PDB 7JTK was used as the starting model with RSP11 and RSP7 mutated to ROPN1l and SPA17 from *D. rerio* respectively. The heterodimer was aligned to PDB 4ZP3 to visualize AKAP18. Asymmetric residues in R2D2 proteins and AKAPs are shown with spheres. **B)** The relative orientation of each heterodimer chain to AKAP18 was switched. **C)** Sequence alignments reveal that AKAP18 and RSP3 are primarily palindromic with matched residues (dots) sharing the same color. The central residues indicated with a highlight are asymmetric. **D)** Alignment of ROPN1L and SPA17 indicating AKAP binding positions with circles and grey highlights. The asymmetric amino acids are highlighted in yellow. **E)** pI values of SPA17 (1-75) and ROPN1L (1-72) from *D. rerio*.

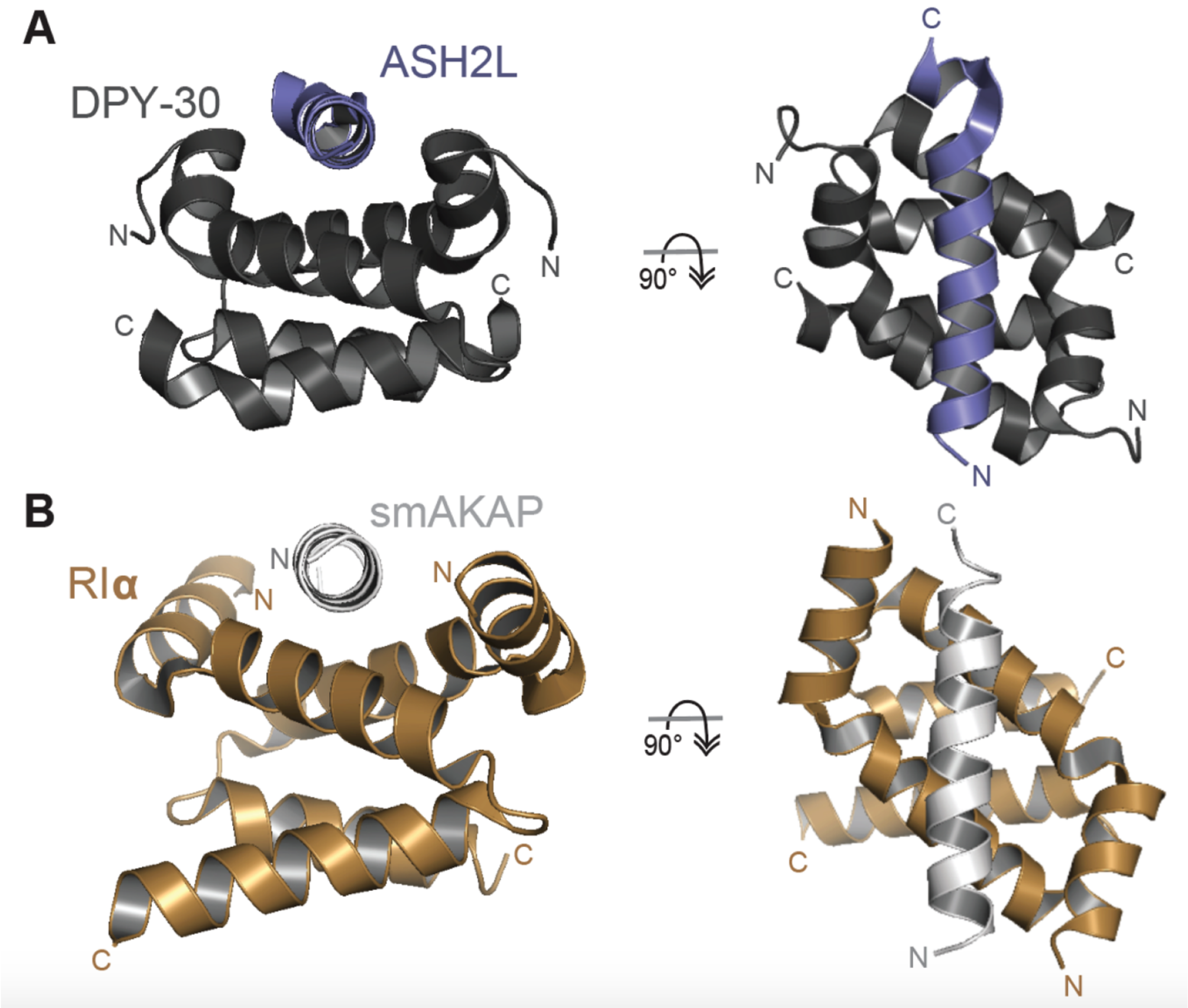

Supplementary Figure S6. Comparison of DPY-30 and RI anchoring interfaces. A) Structure of the DPY-30 (charcoal) in complex ASH2L (purple; PDB. ID =5HVZ). B) Comparison to the R1a (gold)-smAKAP (silver) protein-protein interface (PDB, ID = 4RIQ). Root mean square deviation = 2.9 Å. Top and side views are presented. N and C termini of each polypeptide chain are indicated.
